# Improving research quality: the view from the UK Reproducibility Network institutional leads for research improvement

**DOI:** 10.1186/s13104-021-05883-3

**Published:** 2021-12-20

**Authors:** Andrew J. Stewart, Emily K. Farran, James A. Grange, Malcolm Macleod, Marcus Munafò, Phil Newton, David R. Shanks, the UKRN Institutional Leads

**Affiliations:** 1 University of Manchester, Manchester, UK; 2 University of Surrey, Surrey, UK; 3 Keele University, Keele, UK; 4 University of Edinburgh, Edinburgh, UK; 5 University of Bristol, Bristol, UK; 6 University of Reading, Reading, UK; 7 University College London, London, UK

**Keywords:** Research transparency, Research reproducibility, Research openness, FAIR data

## Abstract

The adoption and incentivisation of open and transparent research practices is critical in addressing issues around research reproducibility and research integrity. These practices will require training and funding. Individuals need to be incentivised to adopt open and transparent research practices (e.g., added as desirable criteria in hiring, probation, and promotion decisions, recognition that funded research should be conducted openly and transparently, the importance of publishers mandating the publication of research workflows and appropriately curated data associated with each research output). Similarly, institutions need to be incentivised to encourage the adoption of open and transparent practices by researchers. Research quality should be prioritised over research quantity. As research transparency will look different for different disciplines, there can be no one-size-fits-all approach. An outward looking and joined up UK research strategy is needed that places openness and transparency at the heart of research activity. This should involve key stakeholders (institutions, research organisations, funders, publishers, and Government) and crucially should be focused on action. Failure to do this will have negative consequences not just for UK research, but also for our ability to innovate and subsequently commercialise UK-led discovery.

## Introduction

Concerns around reproducibility and replicability in research are widespread. In 2021, the UK’s House of Commons Science and Technology Committee launched an inquiry to explore this topic. The UK Reproducibility Network (UKRN) is a consortium of UK Universities aiming to enhance the robustness, transparency, and reproducibility of UK research [1]. As UKRN Institutional Leads, we feel that the discussions that have led to this inquiry reflect a broader need for research transparency, such that all stages of the research pipeline (including research design, data collection processes, the resulting datasets, and analysis code etc.) are made openly available in a manner that allows for re-use. In other words, the ‘replication crisis’ has arisen partly as a result of a lack of research transparency at various stages of the research pipeline, and a lack of incentives at both individual researcher and institutional level to adopt those open and transparent practices.

It is worth noting that concerns about transparency and reproducibility in research (and the role of how researchers are incentivised) are not new [2, 3]. Researchers behave in such a way that is optimal for them in the environment in which they function. Consequently, many engage in behaviours in their work that are most likely to result in reward.

## Main text

### The role of United Kingdom Research and Innovation (UKRI) and other funders

The United Kingdom Research and Innovation (UKRI) policy on open access and the related requirements for outputs in the 2021 Research Excellence Framework (REF) has had a dramatic impact on the proportion of final research outputs (e.g., publications) that are openly available. UKRI and other funders should place a similar strong emphasis on *intermediate* research outputs, involving transparency via full reporting of research workflows, analysis code, and FAIR [4] data, as this is likely to bring about a similar increase in the proportion of more granular research outputs that are open and transparent, and in turn reproducible and that report results that are more likely to be replicable.

Improved transparency will engender greater trust from both the public and the research community, which aligns with the UK Government Research & Development Roadmap [5]. In the same way that UKRI funding councils require grant applications to detail a research data management plan, funders should require researchers applying for funding (not just from UKRI but also from other sources) to develop a detailed plan for how they will ensure the research reported at the point of publication is fully transparent. Consistency across funding bodies and post-award auditing to ensure compliance with respect to this requirement will be important to ensure it has been properly implemented.

In turn, it will be important that the skills required to produce research workflows that are transparent (and ultimately likely to produce findings that are both reproducible and replicable) are fully funded as a component of the project. This could include data curation time, expertise in developing reproducible and transparent research workflows, infrastructure for data curation, and so on. Whilst most of UKRI’s focus on open research has so far been on open access journal articles, UKRI and other funders should place a similarly strong focus on open data, methods, and code that will signify the next stage of UKRI’s open research activity. Crucially, funders must make sure that policies for transparent and open research are accompanied by training and funding.

### Avoiding a ‘One-size-fits-all’ approach

We recognise that for some areas producing research that is open and transparent will be more straightforward than in other areas, and therefore government, funders and institutions should avoid a one-size-fits-all approach to research transparency and ensure research is transparent in a manner that is appropriate for the relevant research discipline and methodology. Mandating that different types of research activity have to be transparent in the same way could result in a lowest common denominator approach, or could turn into a box-ticking exercise. Neither of these is likely to result in the desired outcome and would simply be performative transparency.

Journals and publishers have a role to play in auditing transparency at the point of article peer-review, and researchers and funders during the end-of-research-grant reporting period. It is important that this auditing is done thoroughly to ensure that appropriate transparency in the research workflow has been accomplished. It would be too easy for a researcher to claim their research reporting is transparent when this is not the case. The journal peer review process currently does not typically ensure that the research is reported in a manner that is sufficiently transparent. Indeed, the research literature is full of journal articles that claim the underlying data and analysis code are openly available when this is either not the case, or not delivered in a form that allows them to be (re)usable. Even when data are made available, they can be unusable due to a lack of meta-data and accompanying executable analysis code [6].

When final research outputs (including monographs) are submitted for peer review via the traditional publishing route, it is important that journals and publishers use the review process to ensure that research is reported in a transparent manner, and—if data and analysis code are both provided—that the results can be reproduced. We note that the CODECHECK initiative [7] has the potential to play a key role in this. Researchers submit their analysis code (to https://codecheck.org.uk/) where the team runs the code independently to provide a certificate of executable computation. This approach was used to confirm the COVID modelling work carried out at Imperial College London [8]. Ensuring computational reproducibility should be a standard part of the peer review process.

Research openness and transparency both have a key role to play in innovation and commercialisation. This was highlighted in a recent report by ELIXIR [9] in the context of the Life Sciences in terms of breakthrough discoveries, research excellence and entrepreneurial endeavours that follows from research openness. We recommend engaging with stakeholders in industry to determine the role of research openness and transparency in subsequent innovation and commercialisation.

### Incentive structures

It is important that institutions ensure that organisational structures within which researchers work reward engagement with and adoption of open and transparent research practices. Academic hiring decisions, annual performance reviews, and promotion are often informed by easy-to-calculate research metrics such as the number of research outputs an academic has produced, or the amount of grant income an academic has generated within a particular period. A high score on these metrics does not mean that the underlying research is transparent and robust (often simply that there is a lot of it). Academics need to be incentivised to produce research that is both high-quality *and* transparent.

As competition for academic positions increases, academics are incentivised to behave in a way that will increase their chances of being appointed to a permanent position—which often means focusing on the speed of the research process and the resulting publications, at the expense of attention to openness and transparency. This can encourage a short-term focus on citations and volume. Institutional recruitment and promotion should prioritise and reward conducting research the right way (i.e., with high workflow transparency), rather than getting exciting research (that might have low transparency) published [10]. Reproducible research takes longer, so something needs to be done at the institutional level to raise awareness of this and change assessment criteria accordingly. The publication of research protocols should be recognised as a key component of research transparency. Such publications can be encouraged and rewarded within the existing incentive structures.

In the same way that researchers’ behaviour will change as a result of changes in how those individual researchers are incentivised, universities' behaviours and processes will change only if the ways in which those universities are incentivised changes. If research income (e.g., via research councils and REF Quality Related, QR, income) becomes more dependent on research transparency, then institutional processes with respect to hiring, performance review, and promotion will inevitably adapt to incentivise researchers to adopt transparent practices in their research workflows.

### A team based approach to research and skills development

Many of the computational and data skills needed for researchers to conduct their work in a fully open and transparent manner are lacking in the research community. The 2020 Research & Development Roadmap highlighted a broad lack of digital skills across the UK workforce. In order for UK research activity to remain globally competitive, and to ensure that outputs of that activity are open, transparent, and robust, a joined-up approach across all aspects of R&D (including training) is needed. We are delighted that Research England has provided the UKRN with funding to support our ambitious 5-year project [11] which includes a particular focus on training and the sharing of good practice. We recommend a sustained focus on and investment in digital skills training and infrastructure.

Building open and reproducible research workflows is not a trivial task and often requires researchers to have competence in software coding, data management, etc. We recognise it is an unrealistic goal for researchers to be software engineers in addition to being experts in their discipline. Rather than each individual researcher having the full range of computational and data skills needed for open and reproducible research, we recognise that it is the research team that should have these skills. The days of the ‘lone genius’ as the model of a researcher are fading fast, if not gone already. Therefore, there should be wider support, recognition and reward of team-based research and recognition of the critical role that research software engineers and data stewards play in the research process. As Professor Dame Ottoline Leyser, Chief Executive of UKRI, has said: “We need to build a truly inclusive system that values and nurtures a much wider range of careers and career paths” [12].

### Learning from others

While organisations within the UK are successfully raising awareness in issues around transparency and reproducibility in research, it is important to recognise that other countries are also working in this space, and arguably are further developed in terms of a coherent national research policy. France has recently launched the Second National Plan for Open Science [13] to run from 2021–2024. In 2018 the League of European Universities published an advisory paper [14] detailing a roadmap for change in research culture that captures issues related to transparency and reproducibility under the broader banner of Open Science. The roadmap provides 41 recommendations detailing how this change can be brought about and is built upon the European Commission’s eight ambitions on Open Science [15]. One of these focuses entirely on reproducibility and research integrity. Indeed, the EU has recently produced a scoping report on the topic of reproducibility in research [16].

Networks modelled on the UKRN have been created in other countries, thus providing the opportunity to share knowledge and stimulate a globally-integrated approach to challenges related to research openness and transparency. Activities to encourage international dialogue around a globally integrated approach should be promoted as action that promotes transparency and openness in research must occur not just within any one country, but across the global research community.

It is important that stakeholders develop and put into practice a detailed and financially sustainable long-term and joined-up research strategy focused on openness, transparency, and reproducibility. Failure to do this will have negative consequences not just for research, but also for innovation and subsequent commercialisation of research discovery.

## Outlook

The UK’s House of Commons inquiry that prompted this commentary provides a unique opportunity for the development of an ambitious research vision centred on research openness and transparency that will improve the robustness of research findings, improve public trust in research, maximise the effectiveness and impact of research funding, and provide a strong foundation that places research openness and transparency at the heart of innovation. Individual researchers can support the initiatives we outline above by engaging with organisations that are focused on improving research transparency and openness. They can create their own grassroots activity in this area to work on the different challenges and opportunities that might exist in different disciplines, and can lobby within their institutions to highlight the need to bring about positive change in the academic incentive structure, the on-going provision of researcher training, and the broader culture in which research is conducted.

## Data Availability

Not applicable.

## Abbreviations

FAIR: Findable, Accessible, Interoperable, and Reusable
QR: Quality-related
REF: Research Excellence Framework
UKRI: United Kingdom Research and Innovation
UKRN: UK Reproducibility Network
